# Items, Selections, and Remarks

**Published:** 1870-05

**Authors:** W. W. Miner


### Items, Selections, and Remarks.

BY W. W. MINER, A. B.

Dr. Nolan, lately presented before the Pathological Society of New York, the head of a femur that had been discharged spontaneously from a boy aged fifteen years. The patient was attacked by acute inflammation of the hip joint, which developed into morbus coxarius. Antiphlogistic measures were used and a splint recommended, but the patient refused to wear it. An abscess formed on the outer surface of the thigh, through which at length, the specimen, which had the appearance of epiphyseal separation, was discharged. The patient was doing well.——Dr. Chas. Bliss, of New York, recommends in the *Record* the using of a lachrymal style, formed of three silver wires twisted in the form of a rope.——It is said that burnt alum, inclosed in the vial containing vaccine virus, will aid in its preservation.——Dr. G. W. Murdock, of Cold Spring, N. Y., stated in the *Medical Record* a mode he is using for the internal administration of chloroform. He dissolves one part of chloroform in three parts of glycerine, thus obtaining a solution which is perfectly clear, bland to the taste, and which has but a slight odor of chloroform. In preparing it mix the one part of chloroform with two of the parts of glycerine, adding the former gradually, and carefully rubbing up the mixture; set aside in a corked bottle for twenty-four hours, when a portion of the chloroform will separate and fall to the bottom, and must be removed, and then rubbed up with the third part of the glycerine; the two solutions are then mixed together. The resulting solution is one-eighth less in bulk than the sum of the quantities of the separate ingredients. This preparation probably possesses the advantages afforded by hydrate of chloral, without its expensiveness.

There are fourteen general hospitals in the city of London, and sixty-six special ones. The oldest among these is St. Bartholomew's Hospital, which was first founded in 1123.——A new, spacious and excellent hospital building, under charge of the "Sisters of Mercy," is now completed and being opened in Chicago. Drs. N. S. Davis, H. A. Johnson, E. Andrews, and W. H. Byford have the professional charge of the institution.——The hospital of the National Soldier's Home at Dayton, Ohio, was formally opened on the 20th inst., the Governors of Ohio and Wisconsin participating in the ceremonies.——The State Hospital for Insane at Worcester, Mass., is to have its location changed from the city district to a spacious suburban retreat.——Separate clinics for women are to be provided, henceforth, at the Pennsylvania Hospital.——The next annual meeting of the Ohio State Medical Society will be held at Cleveland, Ohio, on the second Tuesday in June.——The National Association of the Volunteer Medical Officers of the U. S. Army and Navy has just been organized at Washington with C. C. Cox, M. D., L. L. D., of Maryland, as President, and J. B. Hood, M. D., Secretary. The object of the association is the preservation of the names, and an account of the services of its members, their personal recollections and history as medical officers, and the preservation of *esprit du corps.*

M. Claude Bernard is now a member of the Academy of Sciences, and of the French Academy, a senator, a professor at the College of France and at the Garden of Plants, from which positions he derives an annual income of $10,000 in gold.——Prof. Liebig, a German, has received the Albert medal this year, from the Society of Arts, Trade, and Manufactures of Great Britain.——M. Nelaton is said to suffer from heart disease, and to be about to retire from practice.——Profs. Hammond and Echeverria were both witnesses in the McFarland trial at New York, and testified on opposing sides respecting the insanity of the defendant.——Fred. Julius Otto, Professor of Technical Chemistry and

Pharmacy, Germany, well-known to students as an author, died on the 12th of January last.

Aluminium is now obtained by mixing clay with coal tar, and heating the mixture in a retort to redness, while illuminating gas is supplied under a pressure of thirty pounds to the inch, so long as it is absorbed.—The inauguration of this method is a notable success in chemical technics. A simple solution of cyanide of potassium is recommended, on good authority, to be most efficient in the cleansing of tarnished metallic articles.——The *Gas Light Journal* states that "late scientific intelligence from Great Britain expresses the belief that magnesium, by new processes of manufacture, will be brought down to a shilling an ounce retail. At this price, its use in lumps, in the shape of a ribbon, of the thickness of heavy paper, and a tenth of an inch wide, will be decidedly economical."

C. C. Cox, M. D., Professor of Anatomy in Georgetown Medical College, is about to commence the editing a quarterly publication called "*The National Medical Journal.*" The Journal will be published in Washington, and each number is to contain not less than one hundred and twenty-eight octavo pages.——Dr. Wm. Abraham Love, announces that a medical journal entitled the "*Cotton Zone*," is about to be published in Atlanta, Georgia.

Vaccination was made compulsory in Ireland in 1863, and since that period the *Boston Medical Journal*, January, says that small pox has been banished as completely from the island as St. Patrick is said to have banished the snakes.——Dr. Bradford S. Thompson, of New York, in a paper read before the New York Medical Journal Association, states that:—The London cat and dog's meat carriers or sellers—nearly all men—number at least 1,000. It is believed by one who has been engaged in the business for 25 years, that there are from 900 to 1,000 horses, averaging 2 cwt. of meat each, boiled down every week; so that the quantity of cat and dog's meat used throughout London, is about 200,000 pounds per week, and this, sold at the rate of 2½d. per lb., gives £2,000 a week for the money spent in cat and dog's meat, or upwards of £100,000.——Dr. Thompson also states that the amount of beer used in Great Britain and Ireland amounts to 720,200,000 gallons. It is estimated that the quantity of the beverage consumed in Europe each year would require to contain it, a canal five English miles in length, fifty feet deep, and two hundred feet broad. At present England alone, spends $100,000,000 a year in beer, and her expenditures for drugs used in adulterating beer and other intoxicating beverages are enormous.——The *Record* gives an extract from a communication by a Dr. Draper of the *Medical Press and Circular*, which states that ether is now extensively used as an intoxicating beverage in the North of Ireland. The introduction of the practice dates back five years, and its origin is accounted for by one, in the medicinal use of the drug where stimulants are contra-indicated, by another, in the desire of 'getting drunk more cheaply.' The writer says, that with these two exceptions, his authorities are unanimous in the opinion that ether drinking exists in consequence of the laudable efforts of the Roman Catholic clergy in inducing their flocks to abstain from whisky. The quantity of ether taken is from two to four drachms repeated twice, thrice, and even four and six times daily.

Dr. Thudichum has lately given to the French Academy of Science, and the London Chemical Society, an account of a nitrogenous constituent of urine, to which he has given the name of Kryptophanic acid. Of the constituents of urine it is next in abundance to urea, and its formula is $C_{10} H_{18} N_2 O_{10}$. To obtain kryptophanate of lime, the urine is treated with milk of lime, filtered, concentrated by evaporation, slightly acidified with acetic acid, and precipitated with alcohol. This salt is then purified, converted into a lead salt, and the acid set free by the addition of sulphuric acid.——M. Dumas has presented to the French Academy a process devised by Dr. Isaac Adams, of Boston, for the electric deposition of nickel. The process consists in using a solution of a salt of ammonia and nickel, from which the slightest trace of potash or soda is excluded.——It is said that the net-work of railroads and telegraph wires, which exist throughout the country, serves to equalize the electric conditions of the earth and atmosphere, so as to lessen the violence of thunder storms, while a liberal supply of rain is by it brought to regions that were formerly, at certain periods of the year, dry and parched.——The *California Medical Gazette* is publishing, seriatim, a list of the Flora of California, which has been prepared by H. N. Bolander, late State botanist.——A case of poisonsoning by prussic acid vapor occurred in a chemical manufactory in Brussels, by the fracture of a bottle containing 800 grammes of the poison. He was restored by dilute chlorine water and an ammoniacal embrocation. A dog in the room at the time died from the effects of the vapor inhaled.—*Am. Jour. Pharm.* ——The Faculty of a College of Pharmacy has been organized under the auspices of the Chicago Medical College.——Klever, a German chemist, as stated in the *Am. Jour. Pharm.* has found that 100 parts of glycerine will dissolve 50 parts of tannic acid, 33 of sulphate of atropia, 10 of acetate of copper, 30 of sulphate of copper, 1.9 of iodine, 20 of acetate of morphia, 10 of borate of soda, 0.1 of sulphur, etc.——Prof. Boettger observes that oxygen may be obtained at common temperature by using a mixture of the hyperoxides of lead and barium, upon which nitric acid is poured, by which means oxygen free from ozone and antozone is obtained.

We read in the *American Educational Monthly*, that:—Sir Charles Lyell, the great geologist, is remarkable for the reckless eagerness with which he adopts anything that may serve to prop up the theory of an enormous antiquity of the human race. There are a series of elevated beaches on the coast of Scandinavia, which show that the sea once stood several hundred feet higher than it now does, and this rise has taken place since the drift period, or within the time of man. Sir Charles, and others, advance the idea that this rise has been steady and gradual at the rate of two and a half feet in a century thus making the age of man to be at least 240,000 years. Professor Kjerulf, of Christiana, who is making the government geological survey of the coast, and has carefully examined the raised beaches and terraces, declares the whole theory to be utterly baseless. In the first place, he says the uppermost limit of the sea action is only one-tenth as high as Lyell states, and consequently this correction alone would cut down his figures from 240,000 to 24,000 years. Secondly, he proves that the coast has not risen by a constant slow motion, but has a series of sudden elevations, separated by periods of perfect rest; consequently the whole time spent in the elevation may have been very short. This is shown by the abrupt edges of the terraces, rising like stairs, and separated by level areas. Finally, he says that the idea that the coast is now rising is entirely erroneous, and that the present is a stationary period.

